# A comparison of the corneal biomechanics in pseudoexfoliation glaucoma, primary open-angle glaucoma and healthy controls using Corvis ST

**DOI:** 10.1371/journal.pone.0241296

**Published:** 2020-10-26

**Authors:** Zia Sultan Pradhan, Sujit Deshmukh, Shivani Dixit, Shruthi Sreenivasaiah, Sujani Shroff, Sathi Devi, Carroll A. B. Webers, Harsha Laxmana Rao

**Affiliations:** 1 Department of glaucoma, Narayana Nethralaya Eye Hospital, Bangalore, Karnataka, India; 2 University Eye Clinic Maastricht, University Medical Center, Maastricht, The Netherlands; Bascom Palmer Eye Institute, UNITED STATES

## Abstract

**Purpose:**

To compare the corneal biomechanical parameters between pseudoexfoliation glaucoma (PXG), primary open-angle glaucoma (POAG) and healthy controls using Corvis ST.

**Methods:**

A prospective, cross-sectional study was conducted which included 132 treatment-naïve eyes which underwent Corvis ST. The study cohort comprised of 44 eyes with PXG, 42 eyes with POAG and 46 healthy controls. Corneal biomechanical parameters, which included corneal velocities, length of corneal applanated surface, deformation amplitude (DA), peak distance and radius of curvature, were compared between the groups using analysis of variance models.

**Results:**

The 3 groups were demographically similar. The mean IOP was 15.7 ±3 mmHg in the control group, 21.3 ±5 mmHg in the POAG group and 25.8 ±7 mmHg in the PXG group (p<0.0001). Corneal pachymetry was similar across the 3 groups. Mean DA was significantly lower (p<0.0001) in the PXG group (0.86 ±0.18 mm) compared to the POAG group (0.97 ±0.14mm) and the control group (1.10 ±0.15mm). Corneal velocities were also found to be statistically significantly different between the groups. However, after adjusting for IOP, there was no difference in any of the biomechanical parameters between the 3 groups.

**Conclusion:**

Corneal biomechanical parameters measured on Corvis ST are not different between eyes with PXG, POAG and healthy controls after adjusting for IOP.

## Introduction

Corneal biomechanics plays an important role in glaucoma diagnosis and is measured in-vivo using the Ocular Response Analyzer (ORA) and the Corvis ST [1–5]. These devices have been extensively employed to understand the corneal biomechanics in eyes with primary open-angle glaucoma (POAG) [4–9]. Corneal hysteresis (CH) on ORA and deformation amplitude (DA) on Corvis ST were found to be lower in POAG compared to healthy controls [4–7]. However, there is scant literature on the corneal biomechanics in eyes with secondary open-angle glaucoma such as pseudoexfoliation glaucoma (PXG) [10].

PXG is characterized by the deposition of white dandruff-like material within the anterior segment of the eye hampering the aqueous outflow through the trabecular meshwork, and hence resulting in elevated intraocular pressure (IOP). Unlike POAG, which is usually a bilateral disease, two-thirds of PXG patients present with unilateral disease, and the chance of developing glaucoma in the fellow eye is 50% over 15 years [11,12]. PXG also differs from POAG in that it has a distinct histology, a higher IOP which usually does not show an increase in response to topical corticosteroids and it progresses more rapidly [13–15]. These differences indicate that it is a separate entity from POAG.

There are a few studies that have measured the difference in corneal biomechanics between PXG and POAG using ORA but a similar comparison using Corvis ST has not yet been explored [16,17]. Therefore, the purpose of this study was to compare the corneal biomechanical parameters between eyes with PXG, POAG and healthy controls using Corvis ST.

## Materials and methods

This was a prospective, observational study conducted at Narayana Nethralaya, a tertiary eye care centre in Bengaluru, South India between August 2015 to November 2019. The methodology adhered to the tenets of the Declaration of Helsinki for research involving human subjects. Written informed consent was obtained from all participants and the study was approved by the Narayana Nethralaya Ethics Committee. The participants included patients with PXG, POAG and healthy controls.

For the purpose of the study, pseudoexfoliation deposits were defined as [18–20]:

- the presence of whitish flakes on the anterior lens capsule in a typical distribution of a partial/complete peripheral band with or without a central disc, or
- white material deposited on the pupillary border of the iris, or
- uveal stage of pseudoexfoliation (pigments deposited on the anterior lens capsule in a distribution corresponding to the peripheral band with increased pigment in the anterior chamber angle).

Glaucoma was defined as characteristic optic disc changes as determined by glaucoma experts (rim notching, rim thinning, retinal nerve fibre layer defects, disc haemorrhages) with corresponding changes on optical coherence tomography (OCT) or visual fields (VF). Baseline IOP was not used to define glaucoma.

Based on these definitions, the study participants were divided into the following cohorts:

1. Control eyes had normal anterior segment examination (apart from cataract), absence of pseudoexfoliation deposits, intraocular pressure IOP ≤ 21 mmHg and normal posterior segment examination with non-glaucomatous optic discs, as assessed by glaucoma experts.
2. POAG cohort included eyes with open angles on gonioscopy, the absence of pseudoexfoliation deposits in the anterior segment and glaucoma. Baseline IOP was not a criterion used to define glaucoma and this cohort included open-angle glaucoma patients with baseline IOP <21mmHg.
3. PXG cohort included eyes with pseudoexfoliation deposits in the anterior segment and glaucoma.

All participants underwent a comprehensive ocular examination, which included a medical history, slit-lamp biomicroscopy (before and after pupillary dilatation), Goldmann applanation tonometry (GAT), gonioscopy, and a dilated fundus examination. Exclusion criteria were age less than 40 years and eyes with a history of trauma or intra-ocular inflammation. All eyes with history of any ocular surgery were excluded except uncomplicated clear-corneal phacoemulsification done more than 6 months prior to recruitment. All pseudophakic eyes included in the study had undergone an eye examination prior to their cataract surgery at our hospital and those records were reviewed to determine whether pseudoexfoliation deposits were present in their anterior segment. Eyes with any corneal pathology, angle closure disease or retinal pathology were also excluded. Patients with a history of collagen vascular disorders or neurological diseases were not recruited. This was a study on treatment-naïve eyes and patients already on IOP-lowering therapy (eyedrops / laser / surgery) were excluded from the analysis.

All participants underwent an examination with the Corvis ST (Oculus, Wetzlar, Germany) which is a noncontact device that records the entire dynamic reaction of the cornea to a fixed air-impulse. The patient was seated with their head on the chin rest and the air puff nozzle was centered on the eye to ensure correct alignment. The corneal deformation from the air puff was recorded using a high-speed Scheimpflug camera (4330 frames per second) the details of which have been described earlier [1]. The measurements were repeated with a 5-minute delay if there was an error on the video (due to disturbances from eyelashes, eyelids or eye movement) with blank readings displayed for the parameters. Apart from measuring the IOP and the central corneal thickness (CCT), the Corvis ST provides several corneal biomechanical parameters based on the deformation response. The air puff first causes the cornea to move inwards and flatten. At this first applanation phase (A1), the length of the applanated cornea (A1 Length in mm) and the velocity of the corneal apex (A1 Velocity in m/s) are measured. The cornea then continues to move inwards to reach a point of highest concavity. Three biomechanical parameters are measured here. The deformation amplitude (DA in mm) is the total displacement of the corneal apex from the start of deformation to the point of highest concavity. The peak distance (PD in mm) is the distance between the 2 bending points of the concave cornea. The radius of curvature (RC) is the curvature of the central concave cornea. As the cornea begins to assume its normal, convex shape, it passes through the second applanation point (A2) where, again, the length of the flattened cornea (A2 Length in mm) and velocity of the corneal apex (A2 Velocity m/s) are estimated.

All glaucoma patients underwent VF examination using Humphrey Field Analyzer II, model 720i (Zeiss Humphrey Systems, Dublin, CA), with the Swedish interactive threshold algorithm (SITA) standard 24–2 program. OCT imaging of the optic disc and peripapillary region was performed using Cirrus HD-OCT (Carl Zeiss Meditec Inc, Dublin, CA) if media clarity permitted good quality scans.

### Statistical analysis

DA was the primary parameter considered for sample size calculation. Expected mean DAs in the control, POAG, and the PXG groups were considered to be 1.1, 1.05 and 1.0 respectively. The sample size (in an ANOVA design) was therefore calculated to detect a difference of 0.05 in the DA (effect size) between the groups at a power of 80% and an alpha error of 5%. With these assumptions, the sample size was calculated to be 40 eyes in each group.

Statistical analyses were performed using Stata version 14.2 (StataCorp, College Station, Tx) statistical software. Descriptive statistics included mean and standard deviation for continuous variables and percentages for categorical variables. Analysis of variance (ANOVA statistic) was used to evaluate the difference in mean measurements between the 3 cohorts. As measurements from both eyes of the same subject are likely to be correlated, the standard statistical methods for parameter estimation lead to underestimation of standard errors. Therefore, the cluster of data for the study subject was considered as the unit of resampling when calculating standard errors [21]. Analysis of co-variance (ANCOVA) was used to compare corneal biomechanical parameters between groups after adjusting for confounders. An additional ANCOVA analysis was performed using just one eye per patient since inclusion of both eyes may result in a bias. Hence, if both eyes were eligible for the study, one eye was randomly chosen for analysis. A p value of <0.05 was considered statistically significant for the final analysis.

## Results

One hundred and thirty-two eyes of 82 participants were included. The study cohort comprised of 44 eyes with PXG, 42 eyes with POAG, and 46 healthy controls. The 3 study groups were demographically similar as shown in Table 1. The clinical details are shown in Table 2. The refractive status and CCT was similar across the 3 groups. Post hoc comparisons using the Tukey HSD test indicated that the IOP (both Goldmann Applanation Tonometry and Corvis IOP) was significantly different between each of the 3 groups (p<0.01). The cup: disc ratio, Mean Deviation (MD) and Visual Field Index (VFI) were significantly different between the normal and POAG groups (p<0.01) as well as the normal and PXG cohorts (p<0.01), but were similar between the 2 glaucoma groups. The MD was -11.5 ±9 dB in the POAG group and -13.6 ±10dB in the PXG group (p = 0.77). The VFI was 69.1 ±29% in the POAG group and 62.2 ±34% in the PXG group (p = 0.70).

**Table 1:** Demographic data of study participants.

	Control	POAG	PXG	P VALUE^a^
No. of patients	33	29	32	
Mean Age (SD) /years	63.9 (8)	62.0 (8)	65.7 (7)	0.19
No. of males (%)	23 (69.7)	18 (62.1)	21 (65.6)	0.82
No. of Hypertensives (%)	12 (36.4)	9 (31.0)	14 (37.2)	0.59
No. of Diabetics (%)	11 (33.3)	8 (27.6)	9 (28.1)	0.86

POAG: Primary open-angle glaucoma; PXG: Pseudoexfoliation glaucoma; SD: Standard deviation.

^a^ p-value calculated using ANOVA statistic.

**Table 2:** Clinical data of eyes included in study.

	Control	POAG	PXG	P Value^a^
No. of eyes	46	42	44	
Mean BCVA (SD) / logmar	0.16 (0.2)	0.13 (0.2)	0.28 (0.4)	0.04
Mean sphere (SD) / D	0.10 (2.1)	-0.27 (3.6)	0.29 (1.7)	0.59
Mean cylinder (SD) / D	-0.64 (0.8)	-0.69 (0.7)	-0.78 (0.7)	0.67
Mean cup: disc ratio (SD)	0.47 (0.2)	0.75 (0.1)	0.78 (0.1)	<0.0001
No. of pseudophakes (%)	7 (16%)	5 (12%)	4 (9%)	0.65
Mean IOP GAT (SD) /mmHg	15.7 (3)	21.3 (5)	25.8 (7)	<0.0001
Mean IOP Corvis (SD) /mmHg	16.6 (2)	20.1 (4)	24.3 (7)	<0.0001
Mean CCT (SD) /μ	533 (37)	527 (39)	533 (39)	0.67
Mean MD (SD) /dB	-2.9 (4)	-11.5 (9)	-13.6 (10)	0.001
Mean PSD (SD) / dB	2.7 (1)	6.6 (4)	6.4 (3)	0.0008
Mean VFI (SD) / %	94.5 (9)	69.1 (29)	62.2 (34)	0.002

POAG: Primary open-angle glaucoma; PXG: Pseudoexfoliation glaucoma; BCVA: Best corrected visual acuity; SD: Standard deviation; IOP: Intraocular pressure; GAT: Goldmann applanation tonometry; CCT: Central corneal thickness; MD: Mean Deviation; dB: Decibel; PSD: Pattern standard deviation; VFI: visual field index.

^a^ p-value calculated using ANOVA statistic.

The corneal biomechanical parameters as measured on Corvis ST (mean and SD) are shown in Table 3. These were compared between the 3 groups using the ANOVA statistic after statistically adjusting for the use of 2 eyes per patient and the DA, A1 velocity and A2 velocity were found to be significantly different between the groups. The A1 velocity was higher in the control group compared to the POAG and PXG groups suggesting that corneas in eyes with glaucoma were less deformable. The Mean DA was less than 1.0 in the POAG and PXG cohorts implying they had stiffer corneas than controls (where DA was greater than 1.0). The mean DA was significantly different between each cohort (p<0.02). However, previous literature has shown the biomechanics of the cornea is affected by age, CCT and IOP [2,3,22]. The data was hence re-analyzed after adjusting for IOP using the ANCOVA statistic. This revealed that there was no difference in any of the biomechanical parameters between the 3 groups as shown in Table 4.

**Table 3:** Comparison of corneal biomechanical parameters derived from Corvis ST between the 3 groups. All values represent means with standard error in parenthesis.

	Control	POAG	PXG	P Value^a^ (Control vs POAG)	P Value^a^ (Control vs PXG)	P Value^a^ (POAG vs PXG)
A1 length /mm	1.93 (0.04)	2.04 (0.05)	2.02 (0.06)	0.17	0.23	0.83
A1 velocity /ms-1	0.14 (0.004)	0.13 (0.005)	0.12 (0.005)	0.03	0.001	0.19
DA /mm	1.10 (0.03)	0.97 (0.03)	0.86 (0.03)	0.002	<0.001	0.017
Peak distance /mm	4.30 (0.17)	4.15 (0.15)	3.91 (0.17)	0.52	0.11	0.28
Radius of Curvature /mm	7.12 (0.15)	7.17 (0.29)	7.21 (0.24)	0.86	0.75	0.92
A2 length /mm	1.85 (0.07)	1.86 (0.08)	1.99 (0.06)	0.88	0.12	0.20
A2 Velocity/ ms-1	-0.36 (0.02)	-0.30 (0.02)	-0.25 (0.02)	0.038	<0.001	0.06

POAG: Primary open-angle glaucoma; PXG: Pseudoexfoliation glaucoma; A1: Applanation point 1; DA: Deformation amplitude; A2: Applanation point 2.

^a^ P-value calculated using ANOVA statistics after statistically accounting for the use of both eyes per patient in the analysis.

**Table 4:** Comparison of corneal biomechanical parameters derived from Corvis ST between the 3 groups after adjusting for the difference in intraocular pressure. All values represent means with standard error in parenthesis.

	Control	POAG	PXG	P Value^a^ (Control vs POAG)	P Value^a^ (Control vs PXG)	P Value^a^ (POAG vs PXG)
A1 length /mm	2.03 (0.05)	2.04 (0.07)	1.93 (0.06)	0.94	0.24	0.27
A1 velocity /ms-1	0.13 (0.004)	0.13 (0.003)	0.13 (0.004)	0.78	0.48	0.60
DA /mm	0.99 (0.02)	0.98 (0.02)	0.97 (0.02)	0.76	0.63	0.80
Peak distance /mm	3.96 (0.19)	4.19 (0.15)	4.23 (0.17)	0.37	0.33	0.84
Radius of Curvature /mm	7.36 (0.19)	7.15 (0.29)	6.98 (0.24)	0.54	0.25	0.66
A2 length /mm	1.97 (0.07)	1.85 (0.07)	1.87 (0.06)	0.22	0.30	0.82
A2 Velocity/ ms-1	-0.30 (0.02)	-0.31 (0.01)	-0.30 (0.02)	0.83	0.94	0.90

POAG: Primary open-angle glaucoma; PXG: Pseudoexfoliation glaucoma; A1: Applanation point 1; DA: Deformation amplitude; A2: Applanation point 2.

^a^ P-value calculated using ANCOVA statistics after adjusting for the difference in intraocular pressure and statistically accounting for the use of both eyes per patient in the analysis.

An analysis was also performed using only one eye per patient. Therefore, 26 eyes with POAG, 27 eyes with PXG and 29 control eyes were re-analyzed using the ANCOVA statistic (power of this analysis = 0.69). There was no difference in any of the corneal biomechanical parameters between the groups after adjusting for the IOP.

Since pseudoexfoliation syndrome (PXF) is known to be a systemic disorder, the possibility of subclinical pseudoexfoliation in the contralateral eye of unilateral PXF or PXG could not be ruled out. Therefore, an additional ANCOVA analysis was performed after removing all control eyes which had either unilateral PXF or PXG in the fellow eye. Thirty-four control eyes were compared with the POAG and PXG eyes. There was no difference in any corneal biomechanical parameter between the groups after adjusting for the IOP.

## Discussion

The biomechanics of the cornea in glaucomatous eyes has been studied in-vivo using the ORA and, more recently, the Corvis ST [23]. Besides using these devices to discern the effect of corneal tissues on IOP measurements, they have also been used to figure out the role of biomechanics in glaucoma pathogenesis. Most ORA studies included eyes with POAG and showed that the CH was lower in POAG (indicative of a weaker cornea) compared to healthy eyes and this lower CH has also been identified as a risk factor for disease progression [4,5].

There is scant data on the corneal biomechanics (CH and corneal resistance factor, CRF) in PXG using the ORA. Yazgan et al found that the average CH was 3.2 mmHg lower in PXG when compared to healthy controls [24]. However, a drawback of this study was that the corneal pachymetry was significantly different between the groups which could affect the biomechanics. Another drawback was that the PXG patients were not treatment-naïve and most were using prostaglandin analogues for IOP control. Prostaglandin analogues have been shown to cause significant matrix metalloprotein upregulation in the sclera of humans with subsequent extra-cellular matrix degradation and it has been theorized that similar changes could occur in the cornea [25]. A study by Cankaya et al. found the mean CH was significantly lower in PXG eyes (6.9 ± 2.1mmHg) compared to normal controls (9.4 ± 1.4mmHg) with the corneal pachymetry being similar in both groups [26]. However, all PXG patients in this study were on IOP-lowering medications which may have altered the corneal biomechanics of this cohort [26].

A few studies have compared the corneal biomechanics between PXG and POAG using the ORA [16,17]. Ozkok et al found the CH was lower in eyes with PXG (8.8 ± 1.4mmHg) compared to eyes with POAG (9.9 ± 1.2 mmHg; p = 0.0007) despite similar corneal pachymetry [16]. Ayala et al. also showed that the mean CH was 9.8 ± 1.6 mmHg in normal eyes, 9.0 ± 1.9 mmHg in POAG eyes and 8.0 ± 1.5 mmHg in PXG eyes [17]. These differences were statistically significant between the PXG and POAG groups (p = 0.042) and the PXG and normal groups (p = 0.0001), but not between POAG and normal groups [17].

There are no previous studies that have compared the corneal biomechanics between PXG and POAG using Corvis ST. There are several unique biomechanical parameters in the Corvis ST system. A more deformable or weaker cornea is supposed to reach the first applanation point (A1) sooner and have a smaller A1 Length and a higher A1 Velocity; at the point of highest concavity, they have a higher DA, smaller PD and RC; and at the second applanation point they have a smaller A2 Length and lower A2 Velocity [7]. Hence, the present study endeavoured to identify biomechanical differences between the corneas of PXG, POAG and healthy eyes using the Corvis ST. Since only treatment-naïve eyes were included in this study, IOP was an inevitable confounder in our analysis. It is important to note that when IOP was not statistically adjusted for, the PXG and POAG groups (which had significantly higher IOP than the controls) showed a reduced A1 Velocity and DA suggesting these cohorts had stiffer corneas. However, the slower inward velocity and reduced amplitude of deformation of the cornea in these eyes was probably due to the increased IOP resisting the inward corneal movement. After adjusting for IOP, there was no difference in the Corvis ST biomechanical parameters between PXG, POAG and healthy controls. Other studies have also reported that IOP is the strongest predictor of DA, and hence it is imperative to account for it in all analyses of corneal biomechanics using the Corvis ST [27,28].

These results contradict the findings of ORA studies which showed that when compared to healthy controls, corneas of PXG and POAG eyes have a lower CH indicative of a weaker cornea [4]. This disparity is challenging to explain, but it is important to note that these devices make several mathematical assumptions to extract corneal biomechanical information which could affect the parameter measured. Studies which have used both Corvis ST and ORA in the same glaucoma population have found no correlation between some Corvis ST parameters (A1 Velocity and DA) and CH [28]. Other Corvis ST parameters showed a weak correlation with CH compared to the Corneal Resistance Factor (CRF). Additionally, Corvis ST parameters and CRF were significantly correlated with IOP (measured on Goldmann Applanation tonometry) but CH was not [29]. Therefore, although both devices measure the corneal response to an air puff, there are inherent differences between the devices which need to be recognized for a more appropriate interpretation of their measurements. In the ORA, the air pulse is not fixed and continues till the cornea just begins to indent [4,23,30]. In contrast, the Corvis ST has a fixed air puff and if the IOP is greater than 60 mmHg, corneal indentation will not be possible and the machine will be unable to measure the biomechanical parameters [22]. There are also differences in the main biomechanical parameters measured by the ORA and the Corvis ST. The CH of the ORA is determined as the difference between the inward and outward applanation pressures (in mmHg) and it represents the area between the load-unload displacement curve [23]. The DA of the Corvis ST is estimated from the displacement of the corneal apex at rest to the point of highest concavity (in mm) and represents the strain response to a fixed load (the air puff) [22]. Therefore, although both instruments provide corneal biomechanical parameters, these are distinct and not interchangeable. These differences may explain the disparity between our findings and those of pervious ORA studies. The other possible reason for the difference in findings could be that several previous studies included glaucoma patients who were already started on IOP-lowering medication which could potentially weaken the corneal biomechanics of these eyes. Clinical studies of glaucomatous eyes using prostaglandin analogues for prolonged periods have shown lower CH, lower CRF and higher DA as compared to glaucomatous eyes not on these medications after adjusting for IOP [27,30]. A strength of the present study was that only treatment-naïve patients were included and hence the effect of anti-glaucoma medication could not have confounded the results.

A limitation of the present study was the inclusion of diabetic patients as this disease is known to affect the corneal biomechanics [31]. However, the percentage of diabetics in each group was similar and hence this may not have altered the findings. Another drawback was the inclusion of patients who had undergone phacoemulsification, which can alter the corneal structure. However, only eyes which had undergone cataract surgery more than 6 months prior to the study were included to ensure that the corneas were stable since previous studies have shown that corneal biomechanics usually returns to baseline 1 month after cataract surgery [32,33]. Additionally, the percentage of pseudophakic eyes was not significantly different between the groups, and hence, this is unlikely to affect our results. Another issue with the inclusion of pseudophakes was the possibility of missing pseudoexfoliation deposits on the anterior surface of the lens and hence misclassifying eyes. To reduce the chance of this, pseudophakic patients underwent a thorough slit lamp examination to look for deposits on the pupillary margin, a dilated examination for deposits on the remaining anterior capsular rim or on the surface of the posterior chamber intraocular lens and a gonioscopy for deposits in the angle. Although old records were also reviewed for a mention of pseudoexfoliation deposits in the eye, there is a possibility of bias due to missing information. A limitation of the study design was that both eyes of patients were included if eligible which could be a potential confounder of the study results. However, the inclusion of both eyes per patient also has some merits for this study design. For instance, inclusion of the fellow eye of unilateral PXG patients as controls bears the advantage that 2 eyes of the same individual were classified into different cohorts and hence the groups were automatically matched for systemic confounders such as age, diabetic control, etc. Since pseudoexfoliation syndrome is believed to be a systemic condition and, hypothetically, the fellow eye of a unilateral PXG patient may not be “truly normal’, it was included only if it was devoid of any pseudoexfoliation deposits in the anterior segment. Also, an ANCOVA analysis was performed after removing all control eyes which had either unilateral pseudoexfoliation syndrome or PXG in the fellow eye and the results did not change. Additionally, ORA studies have shown that corneal biomechanics is not symmetrical in patients with bilateral POAG and the corneal hysteresis was significantly lower in the eye with the more severe VF loss [34]. In a landmark longitudinal study of 114 eyes of 68 patients, CH and baseline IOP was found to influence the rate of VF progression [5]. Therefore, there is enough evidence to suggest that corneal biomechanics is not uniform between the 2 eyes of an individual and hence inclusion of both eyes will increase the spectrum of each disease evaluated. It is based on these considerations from published literature that the current study was designed with the intention of using both eyes per patient, and a statistical adjustment was made during the analysis to reduce the risk of bias.

## Conclusions

Corneal biomechanical parameters on Corvis ST are not significantly different between eyes with PXG, POAG and healthy controls after adjusting for differences in IOP.

## Supporting information

S1 File This is the file containing all the data used in the manuscript. (XLSX)
